# Contemporary Theory and Research

**Published:** 1893-03-04

**Authors:** 


### CONTEMPORARY THEORY AND RESEARCH.

### INFECTED BULLETS.

Lagarde has conducted a careful research as to whether or not bullets are necessarily sterile. He arrives at the conclusion that the vast majority of cartridges in original packages are sterile and free from septic germs, owing to the thorough disinfection and absolute cleanliness in the process of manufacture. Cartridges out of original packages, however, show micro-organisms upon them, and these are not entirely, if at all, destroyed by the act of firing. This he has ascertained by experiment. When a gunshot wound is inflicted upon a susceptible animal by a projectile infected with anthrax, the animal becomes infected with anthrax, and dies in the vast majority of instances from said infection. Hence it is concluded that the heat developed by the act of firing is not sufficient to destroy all the organic matter on a projectile, notwithstanding the cherished notion of three centuries and more to the contrary.

### COMPRESSED AIR BATH FOR DEAFNESS.

M. Hovent, of Brussels, narrates in the *Revue de Laryngologie* the effect of immersion in the compressed air cabinet on a girl of thirteen, long deaf, and vainly treated by numerous distinguished aurists of Belgium, France, and Germany. Her first bath was accidental—merely as companion of a patient—but the effect was so good that further treatment was pressed, in spite of the opposition of the mother. Aural data are apparently ignored, and the results with the watch test alone are given; the hearing for conversation, as for the watch, returned to the normal in ten days, and was instantly regained when relapses occurred on taking cold later on. The history states that marked gain had followed removal and reduction of the hypertrophic tonsillar tissue of fauces and vault, with persistent catheterisation at Bayer's hands; but all had been speedily lost, and no gain had followed later treatment.

